# Supplementary material for: Distinctive molecular features of regenerative stem cells in the damaged male germline
Source: Nat Commun. 2022 May 6;13:2500. doi: 10.1038/s41467-022-30130-z (PMC9076627; doi:10.1038/s41467-022-30130-z)
Supplement: Supplementary file 1 — Supplementary Information [file 41467_2022_30130_MOESM1_ESM.pdf]

Supplementary information

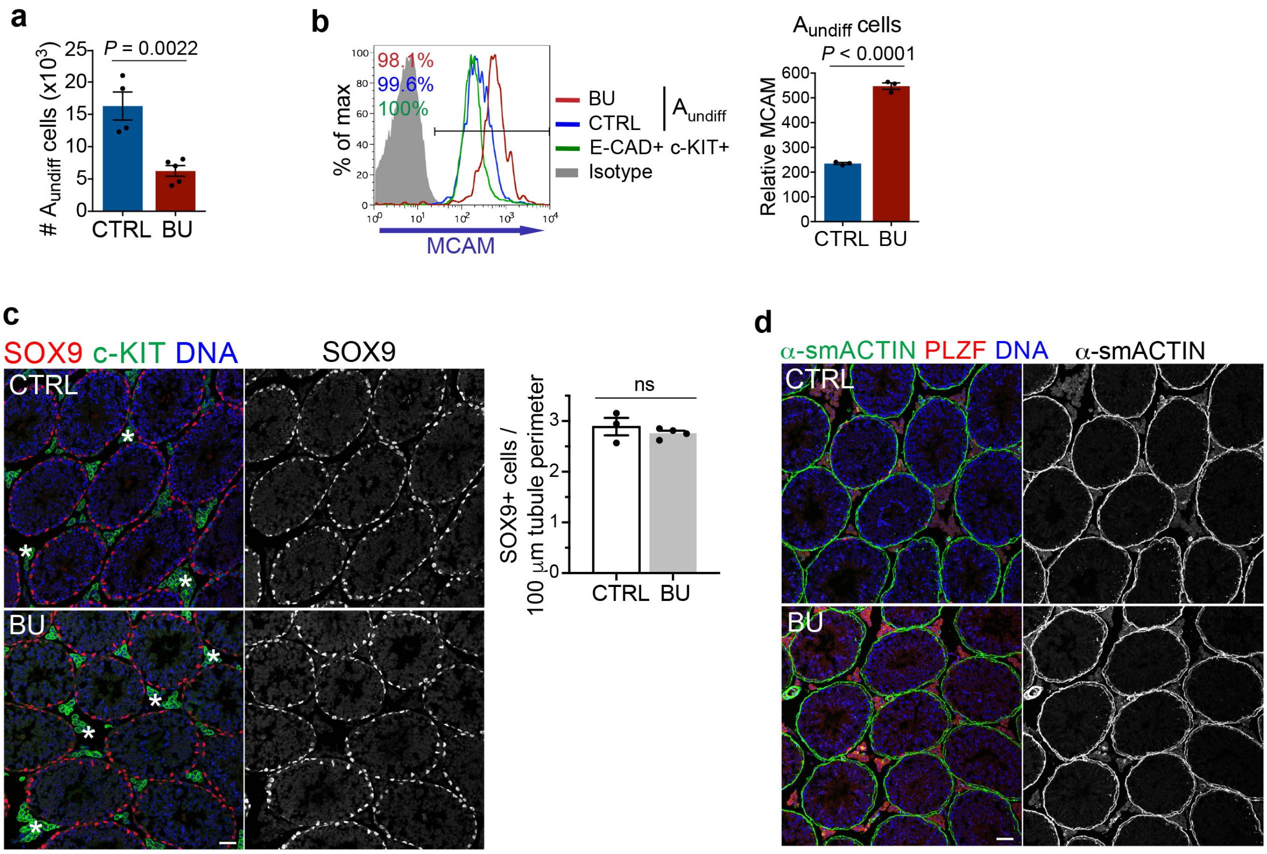

Supplementary Fig. 1 Molecular features of regenerative A_{undiff} following BU treatment. **a** Graph shows number of A_{undiff} (E-Cadherin+ α6-integrin+ c-KIT–) in adult testis D10 post-BU vs. untreated controls from flow cytometry analysis (*n* = 4 per group). **b** Representative flow cytometry of adult WT testis D10 post-BU. MCAM expression in A_{undiff} from control vs. BU-treated mice and A_{diff} (E-CAD+ c-KIT+) of controls is shown in histogram. MCAM is downregulated as A_{undiff} differentiate. Percentages of cells MCAM+ are indicated. Graph shows relative MCAM levels (median fluorescent intensity) on A_{undiff} of control and BU-treated mice (*n* = 3 per group). **c** Representative IF of adult testis sections from control and D10 BU-treated mice for SOX9 as a marker of Sertoli cells. Graph shows number of SOX9+ cells per 100 μm tubule perimeter (*n* = 3 CTRL and *n* = 4 BU, 50 tubule cross-sections scored per animal). Asterisks indicate c-KIT+ Leydig cells within the testis interstitium. **d** Representative IF of adult testis cross-sections from control and D10 BU-treated mice (*n* = 3 per group) for smooth muscle actin as a marker of peritubular myoid cells. Grayscale images in **c**, **d** indicate single-channel staining for somatic markers. Data present as mean ± SEM in **a**-**c**. Significance determined by two-tailed unpaired t-test (ns, $P > 0.05$). Scale bars, 50 μm. Source data are provided as a Source Data file.

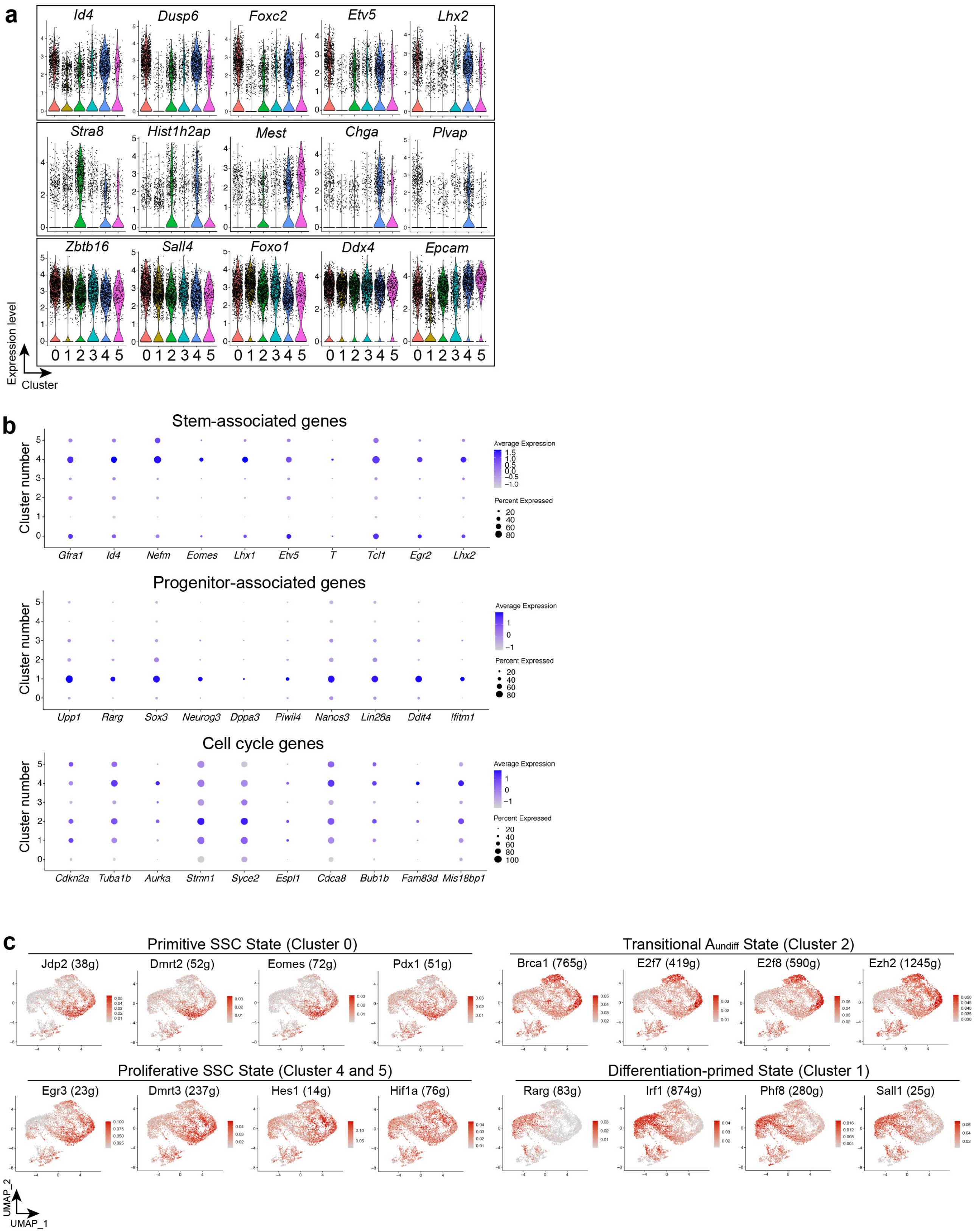

Supplementary Fig. 2 Cluster identity and molecular features of undifferentiated spermatogonia. **a** scRNA-seq data from A_{undiff} (E-Cadherin+ α6-integrin+ c-KIT–) isolated from CTRL and BU-treated mice at D10 was analysed by Seurat. Violin plots show expression of genes that define individual cell clusters (#0-5). Control genes broadly expressed across all clusters are shown in bottom panels. **b** Relative expression of SSC-associated, progenitor-associated and cell-cycle related genes identified from analysis of **a**. Size of dot indicates fraction of cells in clusters positive for gene expression while shade of dot represents average expression level. **c** Gene regulatory network analysis of control samples from single cell dataset of **a** using SCENIC. Selected regulons enriched in each indicated cluster are projected on to UMAP images. Gene (g) numbers in each regulon are indicated.

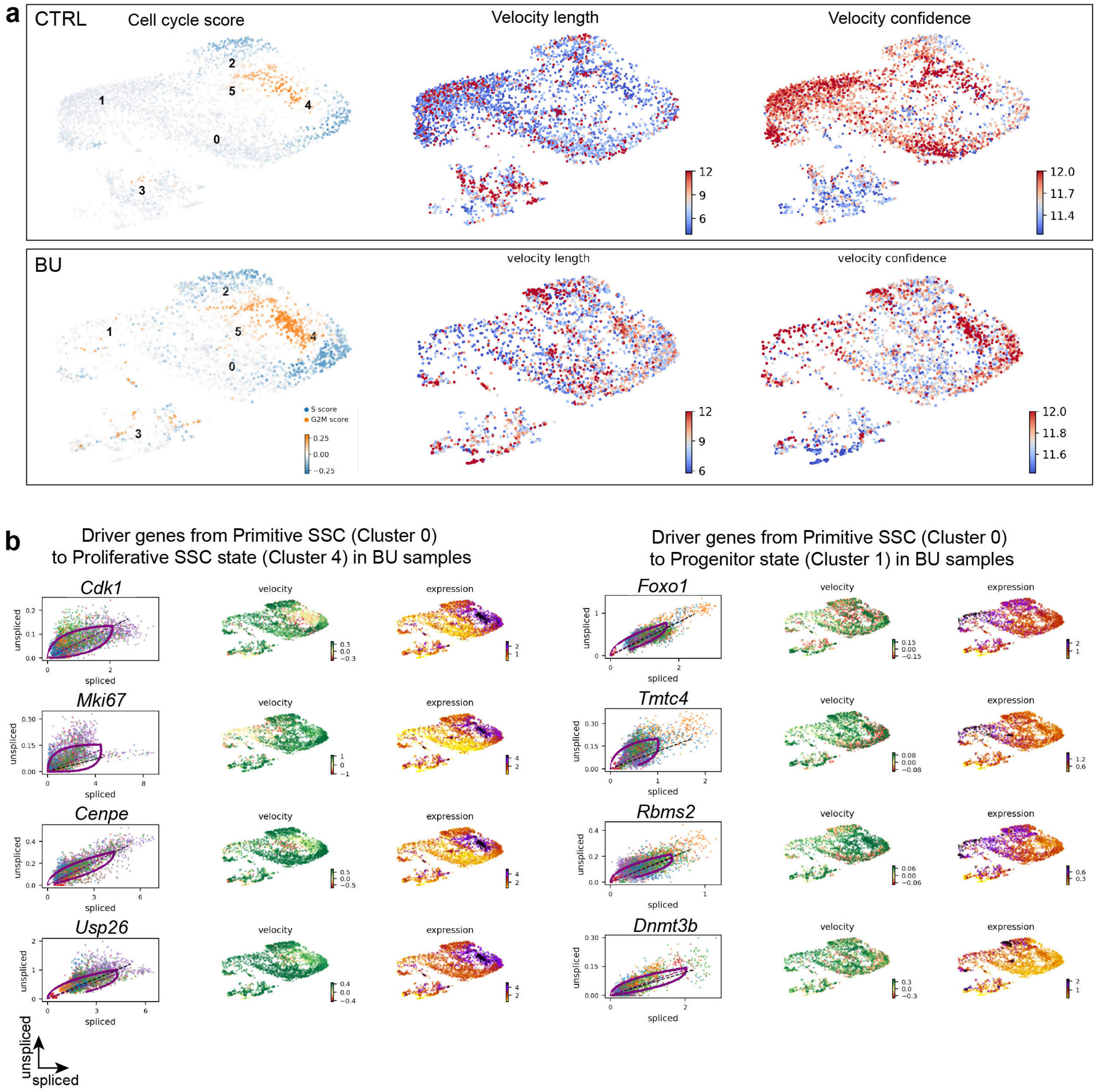

Supplementary Fig. 3 Analysis of homeostatic and regenerative A_{undiff} scRNA-Seq data with scVelo. **a** Cell cycle analysis of control (CTRL) and BU treated samples using a common set of cell cycle genes. Velocity length indicates the speed or rate of cell transitions between clusters, highlighting a faster rate of conversion in BU samples and a more dynamic A_{undiff} population during regeneration. The coherence of neighbouring vectors indicates velocity confidence. **b** Driver gene analysis of BU-treated samples. Top driver genes predicted to promote the transition between the indicated cell clusters during regeneration are shown.

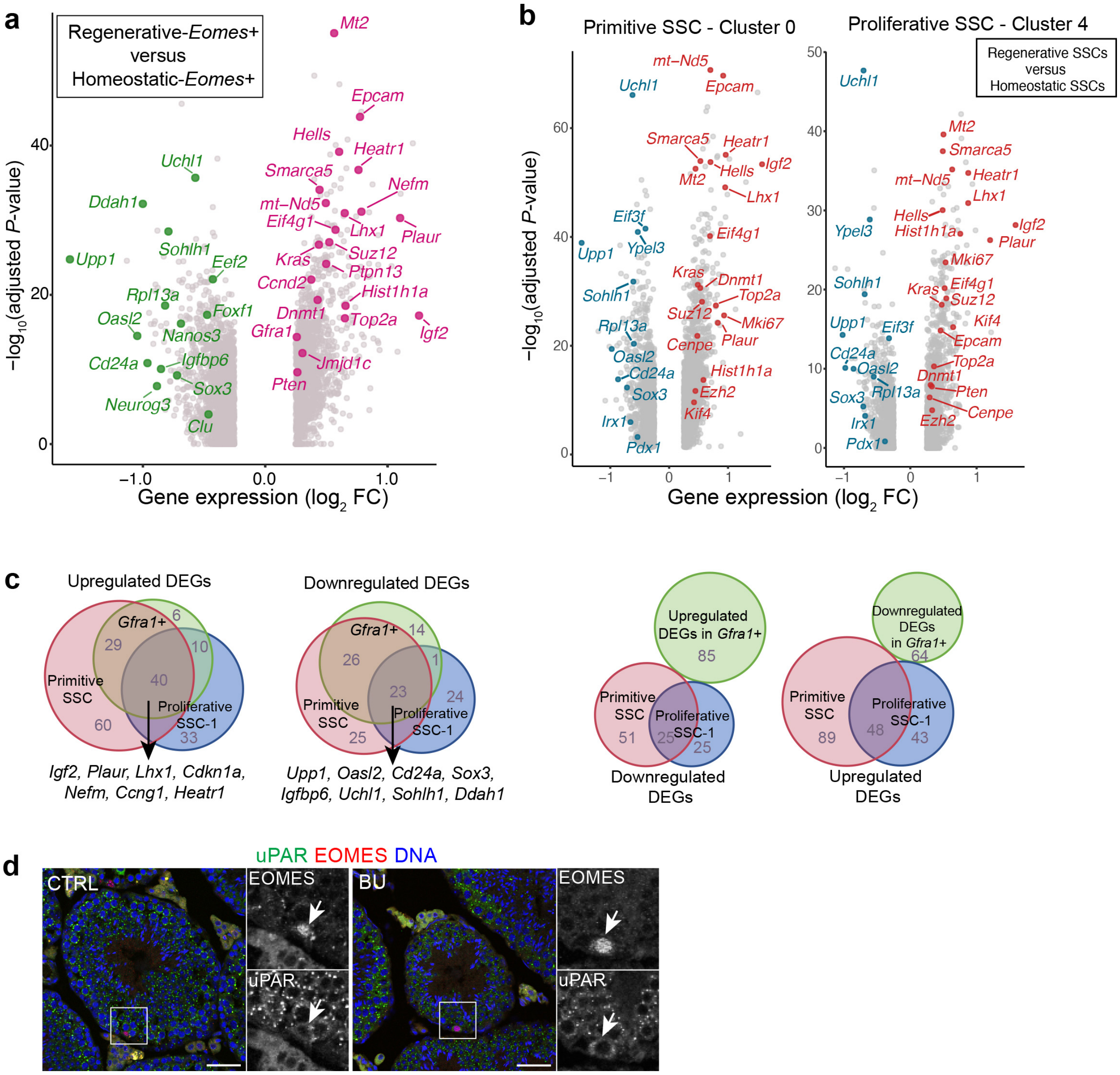

Supplementary Fig. 4 Comparative analysis of regenerative and homeostatic A_{undiff} by single-cell RNA-seq. **a** A_{undiff} isolated from control and D10 BU-treated mice were analysed by scRNA-seq. Volcano plot of DEGs (MAST differential expression test with Bonferroni correction, adjusted *P*-value < 0.05) in *Eomes*+ populations (normalized expression level > 2) is shown. Genes of interest are highlighted. **b** Volcano plot of DEGs (MAST differential expression test with Bonferroni correction, adjusted *P*-value < 0.05) in primitive SSC (cluster 0) and proliferative SSC-1 (cluster 4) populations between CTRL and BU samples from Seurat analysis of scRNA-seq data of **a**. Genes of interest are highlighted. **c** Venn diagrams indicating overlap of concordantly and discordantly regulated DEGs in *Gfra1*+ cells and primitive plus proliferative SSC-1 clusters from scRNA-seq analysis of **a** (Fold change > 1.5 and adjusted *P*-value < 0.05). Selected concordant DEGs between all populations are indicated. **d** Representative IF of testis cross-sections from control and D10 BU-treated mice (*n* = 2 mice per group). Arrows highlight differential uPAR expression in EOMES+ cells. Scale bar: 50 µm.

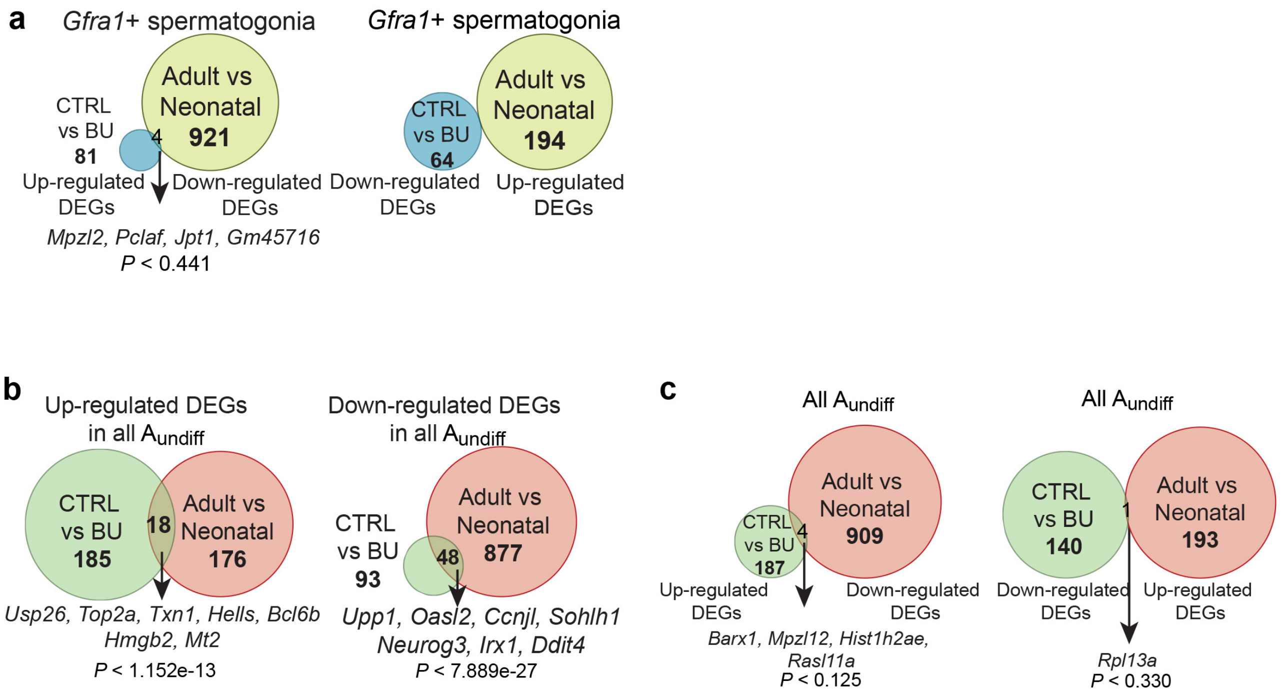

Supplementary Fig. 5 Comparative analysis of A_{undiff} in neonatal, adult and regenerative testis. **a** Venn diagrams illustrating overlap of discordantly regulated DEGs within *Gfra1*+ spermatogonia from scRNA-seq analysis of CTRL vs. BU-treated A_{undiff} and CTRL (adult) vs. neonatal ID4^{bright} spermatogonia. **b-c** Venn diagrams showing overlap of concordantly and discordantly regulated DEGs within A_{undiff} from scRNA-seq analyses of **a**. Examples of concordantly regulated DEGs are indicated in **b**. The few discordantly regulated DEGs identified are shown in **c**. *P*-values are determined by hypergeometric tests in **a-c**.

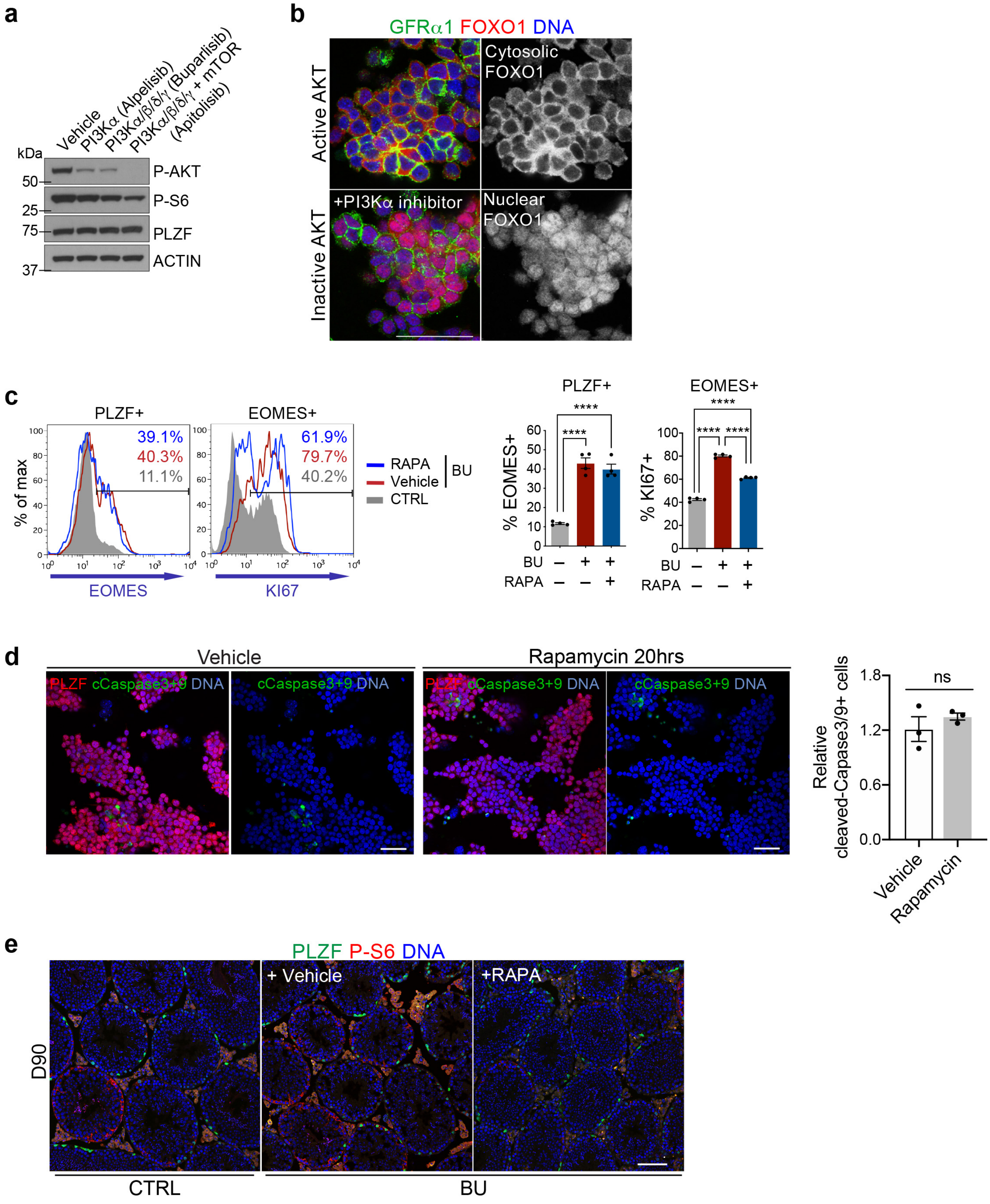

Supplementary Fig. 6 Growth factor-dependent signalling in regenerative A_{undiff}. **a** Representative western blot analysis of WT A_{undiff} cultures treated with selective inhibitor to PI3Kα (alpelisib), a pan-PI3K isoform inhibitor (buparlisib) or dual PI3K/mTOR inhibitor (apitolisib) in complete medium for 3 hours (n = 2 independent cultures). PLZF and ACTIN are used as loading controls. **b** Representative IF of cultured WT A_{undiff} treated with PI3Kα inhibitor or vehicle in complete medium for 1 hour (n = 3 independent lines). Predominant subcellular localisation of FOXO1 and correlation with PI3K/AKT activity are indicated. **c** Representative flow cytometry analysis of testis samples from adult mice treated with BU (10mg/kg) then RAPA or vehicle daily for 7 days from D3 post-BU. Samples were analysed D10 post-BU. Graphs indicate mean percentage of PLZF+ spermatogonia EOMES+ and EOMES+ cells KI67+ ± SEM (n = 4 mice per group). Significance determined by one-way ANOVA followed by Tukey's multiple comparisons test (****P <

0.0001) **d** Representative IF of cultured A_{undiff} treated with vehicle or rapamycin for 20 hours then analysed for apoptotic markers (cleaved Caspase3+9). Graph shows relative number of apoptotic cells normalised to 10000 μm^2 of colony area ± SEM. A minimum of 0.5 mm^2 of colony area was analysed per sample and condition ($n = 3$ independent cultures). Significance was determined by two-tailed unpaired *t*-test (ns, $P > 0.05$) **e** Representative IF of testis cross-sections D90 post-BU from mice treated as in **c** ($n = 3$ mice per condition). Scale bars: 50 μm. Source data are provided as a Source Data file.

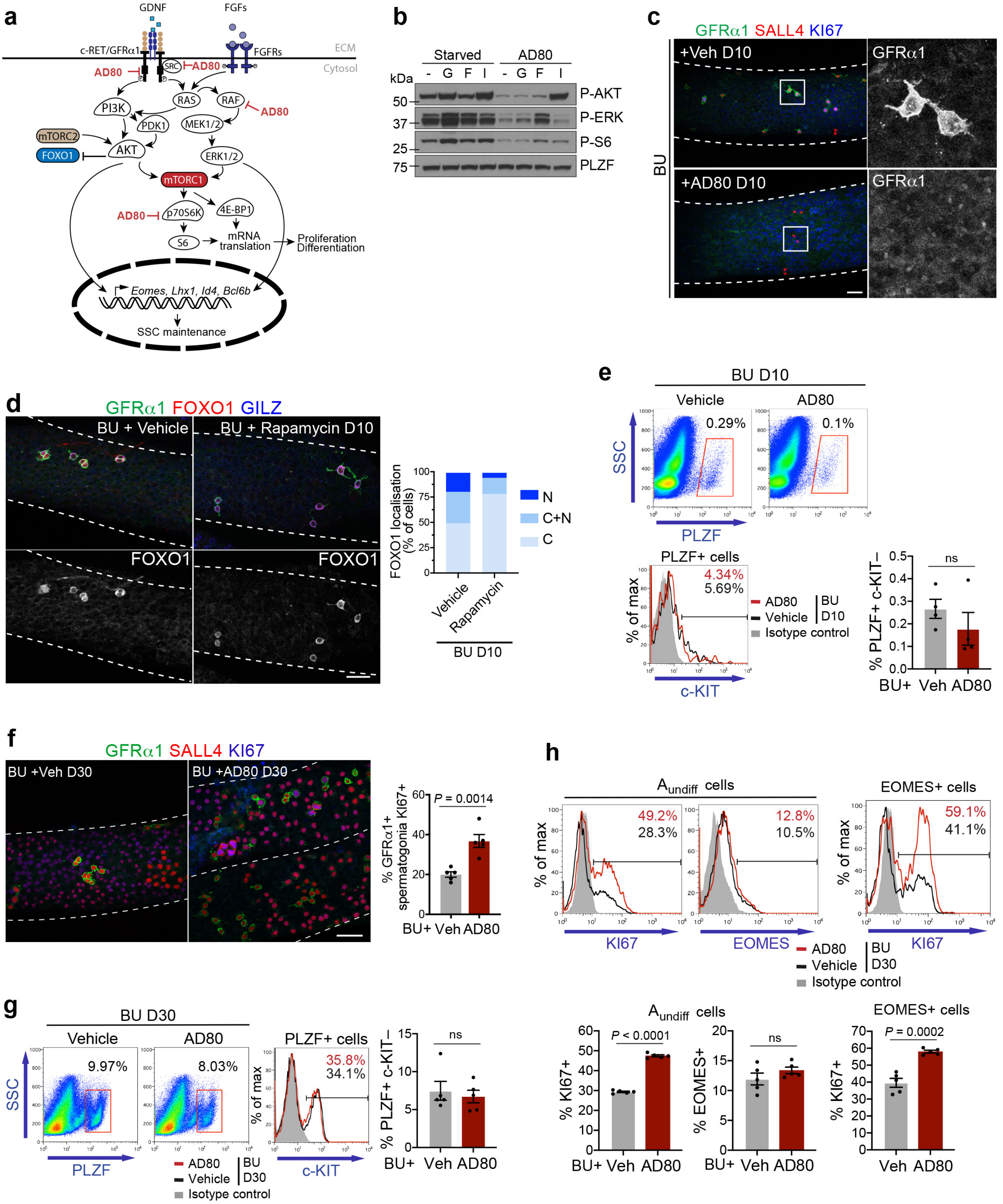

Supplementary Fig. 7 Effects of multikinase inhibitor AD80 on the A_{undiff} regenerative response. **a** Scheme illustrating signalling pathways downstream GDNF and FGFs in A_{undiff} and targets of AD80. **b** WT A_{undiff} cultures were starved for 20h in basal medium lacking cytokines (GDNF, bFGF, EGF and Insulin) and AD80 added for 30 mins prior to stimulation with indicated growth factors (- untreated; G, GDNF; F, bFGF; I, Insulin) for 25 mins and cells harvested. Representative western blot analysis shown (*n* = 3 independent cultures). PLZF is included as loading control. **c** Representative wholemount IF of adult mice treated with BU (10mg/kg) then AD80 or vehicle daily for 5 days from D5 post-BU. Samples were analysed D10 post-BU (*n* = 4 per group). Insets show magnified grayscale images of indicated region. **d** Representative wholemount IF of adult mice treated with BU and then RAPA or vehicle daily for 7 days from D3 post-BU. Samples analysed at D10

post-BU (n = 2 per group, >45mm tubule length analysed per mouse). Graph shows FOXO1 subcellular distribution in the GFRα1+ population (N, predominantly nuclear; N+C, nuclear and cytosolic; C, predominantly cytosolic). **e** Representative flow cytometry of fixed and permeabilised testis cells from mice treated as in **c**. Samples were analysed at D10. Graph shows mean percentage of testis cells A_{undiff} (PLZF+ c-KIT–) ± SEM (n = 4 mice per group). **f** Representative wholemount IF of mice treated as in **c**. Samples were analysed D30 post-BU. Graph shows percentage of GFRα1+ spermatogonia KI67+. Mean values ± SEM shown (n = 5 mice per group). **g**, **h** Representative flow cytometry of fixed and permeabilised testis cells from mice treated as in **c** and analysed at D30. A_{undiff} cells (PLZF+ c-KIT–) and EOMES+ A_{undiff} are shown in **h**. Graph in **g** shows percentage of testis cells A_{undiff} (PLZF+ c-KIT–). Graphs in **h** show percentage of A_{undiff} cells KI67+ and EOMES+ and percentage of EOMES+ A_{undiff} KI67+. Mean values ± SEM shown (n = 5 mice per group). Dashed lines indicate tubule outlines. Scale bars: 50 µm. Significance was calculated by two-tailed Student's t-test in **e-h**. Source data are provided as a Source Data file.

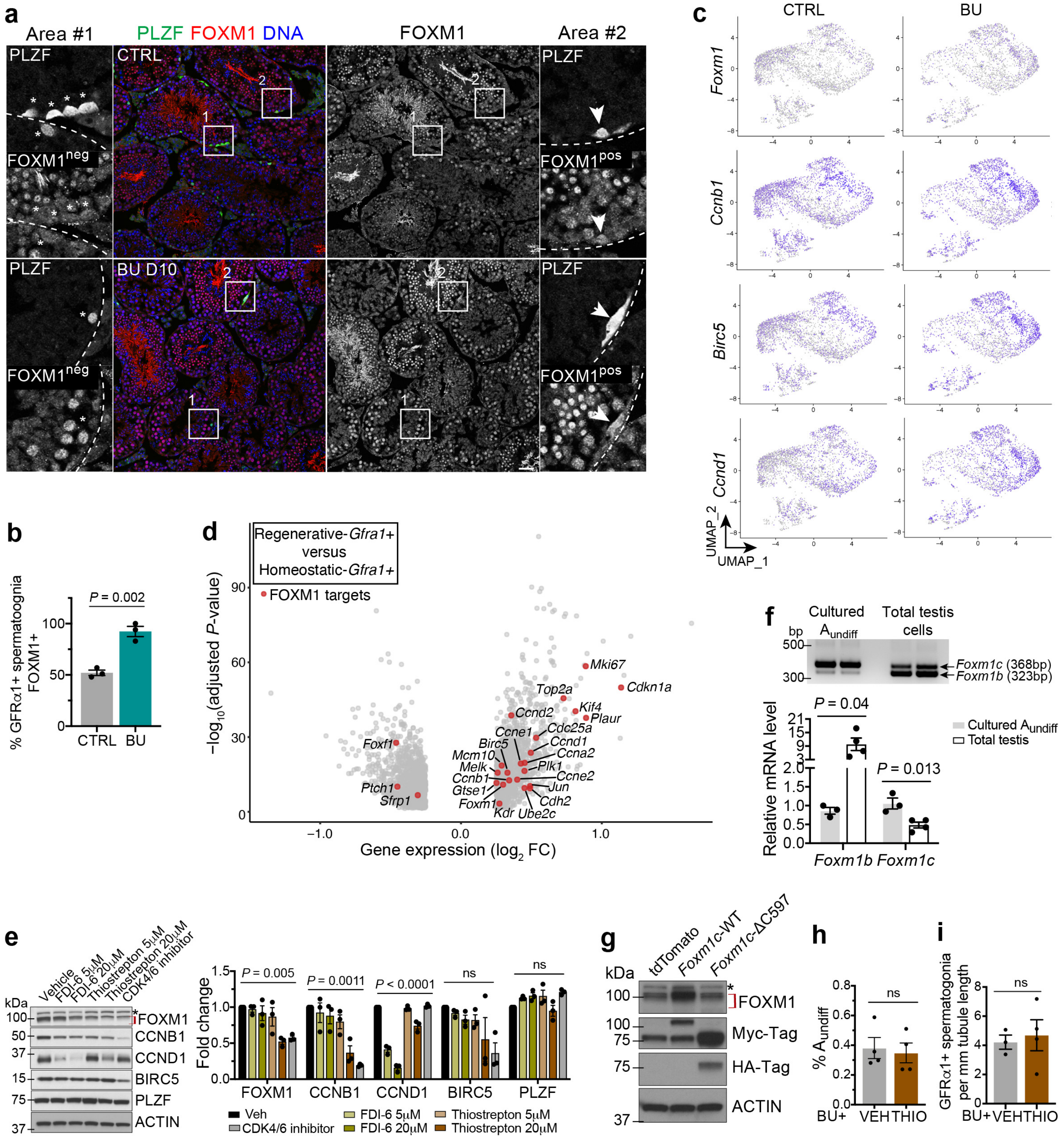

Supplementary Fig. 8 Role of FOXM1 in regulating the regenerative response of A_{undiff} spermatogonia. **a** Representative IF of testis cross-sections at D10 post-busulfan versus controls (CTRL) (n = 3 mice). Insets show higher magnification grayscale images of the numbered areas and illustrate criteria used to define FOXM1 positivity of spermatogonia in CTRL and BU samples. Arrows and asterisks indicate PLZF+ spermatogonia positive and negative for FOXM1 expression respectively. Note that FOXM1 is also detected in meiotic spermatocytes and round spermatids. **b** Graph shows percentage of GFRα1+ cells positive for FOXM1 based on IF analysis of testis cross-sections from adult WT mice D10 after BU vs. untreated controls. Mean values ± SEM shown (n = 3 per group). **c** Expression of *Foxm1* and selected target genes projected on UMAP plot of scRNA-seq analysis of A_{undiff} isolated from control and BU-treated mice at D10. **d** Volcano plot of DEGs (adjusted P-value < 0.05, MAST differential expression test with Bonferroni correction) in *Gfra1*+ (normalized expression level > 2) populations from scRNA-Seq analysis of A_{undiff} isolated from control and D10 BU-treated mice. FOXM1 target genes are highlighted. **e** Representative western blot of WT A_{undiff} cultures treated with FOXM1 inhibitors FDI-6 and thiostrepton at indicated concentrations in complete media for 20 hours. Red bracket indicates FOXM1 protein while asterisk indicates non-specific band. Graph shows protein levels corrected to ACTIN and normalised to vehicle-treated cells. Mean values ± SEM are shown (n

= 3 independent cultures). **f** Analysis of *Foxm1* isoform expression in cultured A_{undiff} and unfractionated adult testis by representative RT-PCR (top panel; 2 independent cultures and testes) and RT-qPCR (graph; *n*=3 per sample type). Sizes of expected amplicons are indicated on gel. Expression levels are corrected to β-actin in RT-qPCR and normalized to control sample. Mean values ± SEM shown in graph. **g** Representative western blot analysis of WT A_{undiff} cultures transduced with *Foxm1c* and *Foxm1c*-ΔC597 lentiviral constructs to confirm FOXM1 antibody specificity (*n* = 3 independent cultures). Red bracket indicates full-length FOXM1 protein while asterisk indicates non-specific band generated by FOXM1 antibody on western blot. **h** Graph shows percentage of testis cells that are A_{undiff} (PLZF+ c-KIT–) from flow cytometry analysis of D10 BU-treated mice treated with thiostrepton or vehicle daily from D3 to D9. Mean values ± SEM shown (*n* = 4 mice per group). **i** Graph shows abundance of GFRα1+ spermatogonia from wholemount IF of mice treated as in **h**. Mean values ± SEM shown (*n* = 3 vehicle treated mice and *n* = 4 thiostrepton treated mice). Significance was determined by one-way ANOVA followed by Tukey's multiple comparisons test in **e** and two-tailed unpaired *t*-test in **b**, **f**, **h**, **i** (ns, $P > 0.05$). Source data are provided as a Source Data file.

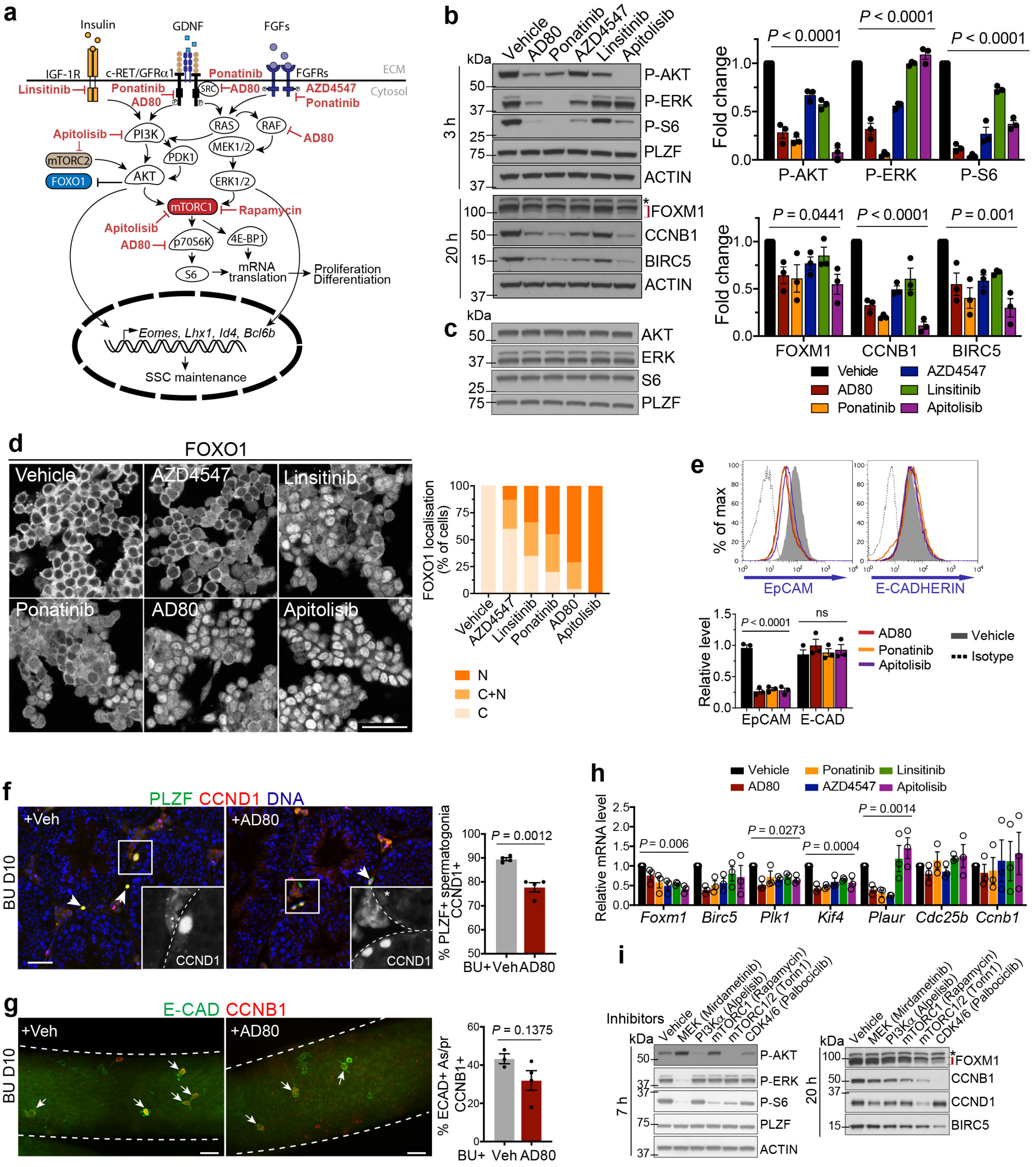

Supplementary Fig. 9 Growth factor-dependent signalling and A_{undiff} regenerative responses. **a** Scheme illustrating growth factor signalling pathways in A_{undiff} and targets of indicated inhibitors. **b**, **c** Representative western blots of WT A_{undiff} cultures treated with kinase inhibitors shown in **a** or vehicle in complete medium. Signalling proteins were analysed after 3h of treatment while changes in FOXM1 and targets after 20h. PLZF and ACTIN were used as loading controls. Red bracket indicates FOXM1 protein while asterisk indicates non-specific band. Associated graphs show protein levels corrected to ACTIN and normalised to vehicle-treated cells. Mean values ± SEM shown (n = 3 independent cultures). Levels of indicated signalling proteins are unaffected by inhibitor treatment as shown in **c**. **d** Representative IF of cultured WT A_{undiff} treated with the indicated inhibitor in complete media for 1hr. Graph shows FOXO1 subcellular distribution (N, predominantly nuclear; N+C, nuclear and cytosolic; C, predominantly cytosolic) determined from a minimum of 250 cells per sample per treatment group (n = 3 independent cultures) **e** Representative flow cytometry analysis of EpCAM and E-Cadherin expression in WT A_{undiff} cultures treated with indicated kinase inhibitors in complete medium for 20h. Graph shows mean relative levels of EpCAM and E-CADHERIN (median fluorescent

intensity) ± SEM (n = 3 independent cultures). **f** Representative IF analysis of testis sections from mice treated with BU (10mg/kg) then AD80 or vehicle daily for 5 days from D5 post-BU. Samples were analysed D10 post-BU. Insets show higher magnification grayscale images of CCND1 in indicated regions. Arrows: CCND1+ PLZF+ spermatogonia. Asterisk: CCND1 − PLZF+ spermatogonium. Dashed lines indicate tubule basement membrane. Graph shows mean percentage of PLZF+ spermatogonia CCND1+ ± SEM (n = 4 mice per group). **g** Representative wholemount IF of adult mice treated as in **f**. Arrows indicate E-CADHERIN+ A_s and A_{pr} CCNB1+. Dashed lines show tubule outline. Graph shows mean percentage of E-CAD+ A_s/A_{pr} CCNB1+ ± SEM and associated P value (n = 3 vehicle, n = 4 AD80 mice). **h** RT-qPCR analysis of WT A_{undiff} cultures treated with indicated inhibitors for 20h in complete medium. Expression levels are corrected to β-actin and normalized to control sample. Mean values ± SEM shown (n = 3 independent cultures). **i** Representative western blot analysis of WT A_{undiff} cultures treated with indicated inhibitors or vehicle in complete medium. Torin is an mTOR kinase inhibitor that suppresses both mTORC1 and AKT signalling. Signalling proteins were analysed after 7hr of treatment while changes in FOXM1 and targets after 20h. PLZF and ACTIN were used as loading controls. (n = 3 independent cultures). Red bracket indicates FOXM1 while asterisk indicates non-specific band. Scale bars 50µm. Significance was determined by one-way ANOVA followed by Tukey's multiple comparisons test in **b, e, h** and two-tailed unpaired t-test in **f**, **g** (ns, $P > 0.05$). Source data are provided as a Source Data file.

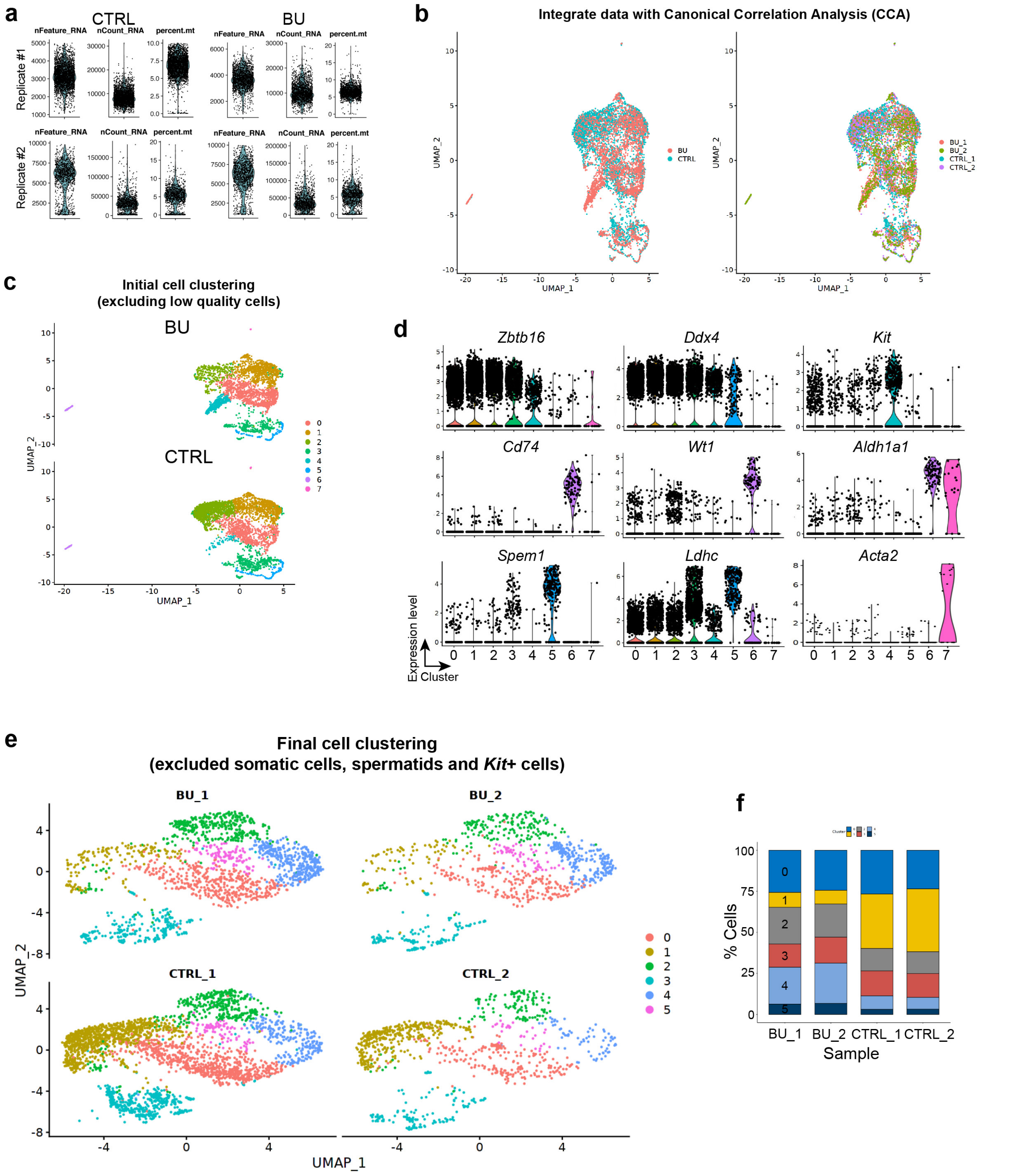

Supplementary Fig. 10 Overview of Seurat-based processing workflow for scRNA-Seq datasets. **a** Violin plots showing QC features of all cells in each sample of 2 experimental replicates. **b** Data integration using Canonical Correlation Analysis (CCA) with batch effect correction. Merged (left panel) and individual samples (right panel) are shown. **c** Initial clustering analysis of all cells from **a** excluding low-quality cells. Cells with >1000 genes expressed and <20% of reads mapped to the mitochondrial genome were retained. **d** Violin plots show expression of genes defining individual cell clusters (#0-7). Clusters 0-4 were identified as spermatogonia (*Zbtb16*+, *Ddx4*+), cluster 5 as spermatids (*Spem1*+), and clusters 6 and 7 as contaminating somatic cells (*Cd74*/*Wt1*/*Aldh1a1*/*Acta2*+). **e** Clustering analysis of each sample and replicate excluding *Kit*+ spermatogonia and contaminating spermatids and somatic cells. Cells combined from each condition were used in subsequent analyses. **f** Graph shows distribution of cells from CTRL and BU-treated mice of each experimental replicate into cell clusters identified in **e**. Similar distributions were observed in replicate samples.

Supplementary Table 1 Primer sequences for RT-qPCR.

Gene	Primer sequence	
	**Forward**	**Reverse**
Actb	GGCTGTATTCCCCTCCATCG	CCAGTTGGTAACAATGCCATGT
Birc5	GAGGCTGGCTTCATCCACTG	CTTTTTGCTTGTTGTTGGTCTCC
Ccna2	TGGATGGCAGTTTTGAATCACC	CCCTAAGGTACGTGTGAATGTC
Ccnb1	CTTGCAGTGAGTGACGTAGAC	CCAGTTGTCGGAGATAAGCATAG
Ccnd1	GTTCATTTCCAACCCACCC	CTCAGATGTCCACATCTCGC
Ccne1	GTGGCTCCGACCTTTCAGTC	CACAGTCTTGTCAATCTTGGCA
Cdc25b	TCCGATCCTTACCAGTGAGG	GGGCAGAGCTGGAATGAGG
Foxm1	ATCGCTACTTGACATTGGACCA	GATTGGGTCGTTTCTGCTGTG
Kif4	ATTCAATGGGAGGTGCATACAC	ACGAGATGAACATAGAAGGTCCA
Plaur	GACTACCGTGCTTCGGGAATG	ATGGTCCTGTTGGTCTTTTCG
Plk1	CTTCGCCAAATGCTTCGAGAT	TAGGCTGCGGTGAATTGAGAT
Zbtb16	CTCCGTAAGCGTCCCCTCTGC	GGTGCAGGCTAGCACCGTCC
