# Supplementary material for: Distinctive molecular features of regenerative stem cells in the damaged male germline
Source: Nat Commun. 2022 May 6;13:2500. doi: 10.1038/s41467-022-30130-z (PMC9076627; doi:10.1038/s41467-022-30130-z)
Supplement: Supplementary file 3 — Description of Additional Supplementary Files [file 41467_2022_30130_MOESM3_ESM.docx]

**Description of Additional Supplementary Files**

**Supplementary Data 1** Analysis of gene expression in regenerative vs. homeostatic A_undiff_. (Excel file). The A_undiff_ population (E-Cadherin+ c-KIT– α6-integrin+) was isolated from untreated control and BU-treated mice at D10 for RNA-seq analysis (*n* = 4 per group). Table includes differentially-expressed genes (DEGs) in regenerative vs. control (homeostatic) A_undiff_ (false discovery rate < 0.05, absolute fold change > 1.5, Limma-Voom using empirical Bayes method).

**Supplementary Data 2** Differentially expressed genes within each identified cell cluster from scRNA-Seq analysis of A_undiff_ spermatogonia (E-Cadherin+ c-KIT– α6-integrin+) isolated from control and BU-treated mice at D10 (3 mice pooled per group, 2 independent experimental repeats) (Excel File). Cut-off for DEGs is adjusted *P*-value < 0.05 (MAST differential expression test with Bonferroni correction). Second sheet includes top 10 cluster-specific regulons identified from SCENIC analysis of control sample.

**Supplementary Data 3** Differentially-expressed genes in SSC-enriched fractions of regenerative and homeostatic A_undiff_. (Excel file). A_undiff_ spermatogonia (E-Cadherin+ c-KIT– α6-integrin+) were isolated from untreated control and BU-treated mice at D10 and analysed by scRNA-seq (cells from 3 mice pooled per group, 2 independent experimental repeats). Cells positive for expression of SSC-associated genes *Gfra1* or *Eomes* (normalised expression level >2) or present within primitive SSC (cluster 0) and proliferative SSC-1 (cluster 4) clusters from Seurat analysis were selected from the A_undiff_ population. Tables show DEGs in regenerative vs. homeostatic *Gfra1*+ and *Eomes*+ fractions and indicated SSC clusters (adjusted *P*-value < 0.05, MAST differential expression test with Bonferroni correction).

**Supplementary Data 4** Comparative analysis of gene expression in A_undiff_ during regeneration and development. (Excel file). Table shows concordantly regulated DEGs identified from scRNA-seq analysis of adult homeostatic vs. regenerative A_undiff_ (this study) and neonatal ID4^bright^ cells^36^. Table includes concordant DEGs within total A_undiff_ populations and *Gfra1*+ SSC-enriched fractions (adjusted *P*-value < 0.05, normalised expression level > 2, MAST differential expression test with Bonferroni correction). Overlapping DEGs (adjusted *P*-value < 0.05 and fold change > 1.5) similarly up- or down-regulated in both datasets are included as separate sheets.

**Supplementary Data 5** Pathway analysis of differentially expressed genes (DEGs) in regenerative A_undiff_. (Excel file). DEGs identified from bulk RNA-seq analysis of A_undiff_ populations isolated from BU-treated adult mice at D10 vs. untreated controls were processed by Ingenuity Pathway Analysis (IPA). Table shows predicted upstream regulators of the regenerative A_undiff_ state plus associated Activation Z-Scores and identified genes in the different pathways. Predicted activation state of upstream regulators is included. Overlapping *P*-values were calculated in IPA using a one-sided Fisher’s Exact test.

**Supplementary Data 6** Antibodies and inhibitors used in the study. (Excel file). List of antibodies and dilutions used for IF, flow cytometry and western blot. Second sheet includes details of inhibitors used, specific targets, source and reported IC_50_ values.

**Supplementary Data 7** Quality control details for scRNA-Seq analysis (Excel File). Sequencing metrics from the Cell Ranger pipeline for each sample and experimental replicate. Second sheet includes numbers of cells from initial Seurat clustering analysis of control and BU-treated samples following filtering steps to remove low quality cells.
