# Supplementary material for: Distinctive molecular features of regenerative stem cells in the damaged male germline
Source: Nat Commun. 2022 May 6;13:2500. doi: 10.1038/s41467-022-30130-z (PMC9076627; doi:10.1038/s41467-022-30130-z)
Supplement: Supplementary file 11 — Reporting Summary [file 41467_2022_30130_MOESM11_ESM.pdf]

Corresponding author(s): Tin Lap Lee and Robin M Hobbs
Last updated by author(s): Mar 1, 2022

Reporting Summary

Nature Portfolio wishes to improve the reproducibility of the work that we publish. This form provides structure for consistency and transparency in reporting. For further information on Nature Portfolio policies, see our Editorial Policies and the Editorial Policy Checklist.

Statistics

For all statistical analyses, confirm that the following items are present in the figure legend, table legend, main text, or Methods section.

n/a	Confirmed	
☐	☒	The exact sample size (*n*) for each experimental group/condition, given as a discrete number and unit of measurement
☐	☒	A statement on whether measurements were taken from distinct samples or whether the same sample was measured repeatedly
☐	☒	The statistical test(s) used AND whether they are one- or two-sided<br>*Only common tests should be described solely by name; describe more complex techniques in the Methods section.*
☒	☐	A description of all covariates tested
☐	☒	A description of any assumptions or corrections, such as tests of normality and adjustment for multiple comparisons
☐	☒	A full description of the statistical parameters including central tendency (e.g. means) or other basic estimates (e.g. regression coefficient) AND variation (e.g. standard deviation) or associated estimates of uncertainty (e.g. confidence intervals)
☐	☒	For null hypothesis testing, the test statistic (e.g. *F*, *t*, *r*) with confidence intervals, effect sizes, degrees of freedom and *P* value noted<br>*Give P values as exact values whenever suitable.*
☒	☐	For Bayesian analysis, information on the choice of priors and Markov chain Monte Carlo settings
☒	☐	For hierarchical and complex designs, identification of the appropriate level for tests and full reporting of outcomes
☒	☐	Estimates of effect sizes (e.g. Cohen's *d*, Pearson's *r*), indicating how they were calculated

Our web collection on statistics for biologists contains articles on many of the points above.

Software and code

Policy information about availability of computer code

Data collection	Flow cytometry: FACS DIVA 6.0<br>Microscopy: Zeiss LSM780 FCS software, Nikon C1 software, Olympus Stereologer software<br>qRT-PCR: Mic qPCR Cycler software<br>RNA-Seq: Illumina NextSeq and HiSeq software.
Data analysis	FlowJo 8.7, Prism 8.0, Ingenuity Pathway Analysis (Qiagen), R v3.6; python 3.7.3; cellRanger v3.1; velocyto v0.17.17; R packages (Seurat v3.2.1; SCENIC v1.1.3; clusterProfiler v3.14.3; tidyverse v1.3.0; AUCell v1.8.0; MAST v1.12.0; Augur v1.0.0); python packages (scvelo 0.2.2; scanpy 1.6.0; scikit-learn v0.23.2; scipy v1.5.1); custom R and python scripts.<br>For the bulk RNA-seq analysis, R packages include limma v3.40.6, edgeR v3.26.8, jsonlite v1.7.2, ggplot2 v3.3.5 and ComplexHeatmap v2.2.0. Degust code for analysis of bulk RNA-Seq data is provided in a Zenodo repository (https://doi.org/10.5281/zenodo.3258932). Seurat for scRNA-Seq analysis is available from GitHub (https://github.com/satijalab/seurat/). SCENIC and scVelo are available from https://scenic.aertslab.org/ and https://scvelo.org respectively.

For manuscripts utilizing custom algorithms or software that are central to the research but not yet described in published literature, software must be made available to editors and reviewers. We strongly encourage code deposition in a community repository (e.g. GitHub). See the Nature Portfolio guidelines for submitting code & software for further information.

Data

Policy information about availability of data

All manuscripts must include a data availability statement. This statement should provide the following information, where applicable:

- Accession codes, unique identifiers, or web links for publicly available datasets
- A description of any restrictions on data availability
- For clinical datasets or third party data, please ensure that the statement adheres to our policy

The bulk and single cell RNA-Seq data generated in this study have been deposited in the Gene Expression Omnibus (GEO) database under accession codes GSE182727 (https://www.ncbi.nlm.nih.gov/geo/query/acc.cgi?acc=GSE182727) and GSE182924 (https://www.ncbi.nlm.nih.gov/geo/query/acc.cgi?acc=GSE182924) respectively. The published single cell RNA-Seq dataset of neonatal Aundiff used in this study is available under accession code GSE109049 (https://www.ncbi.nlm.nih.gov/geo/query/acc.cgi?acc=GSE109049) (PMID: 30404016). Source data underlying figures are provided with this paper.

Field-specific reporting

Please select the one below that is the best fit for your research. If you are not sure, read the appropriate sections before making your selection.

☒ Life sciences ☐ Behavioural & social sciences ☐ Ecological, evolutionary & environmental sciences

For a reference copy of the document with all sections, see nature.com/documents/nr-reporting-summary-flat.pdf

Life sciences study design

All studies must disclose on these points even when the disclosure is negative.

Sample size	No statistical method was used to predetermine sample sizes.<br>For quantitative analysis of testis samples a standard minimum of 3 biological replicates (individual mice) were analysed per group and time point. Sample sizes were based on prior experience (PMID: 31123254; 30026551) and sufficient to obtain statistical significance.
Data exclusions	No data were excluded except in single cell RNA-seq analysis when contaminating cell types were excluded based on marker gene expression as detailed in respective methods section.
Replication	Details on experiment replication are included in the Figure legends.
Randomization	No specific randomization methods were used. Age matched wildtype male mice were randomly allocated into specific cages by animal staff prior to the experiment and cages were randomly assigned into individual experimental groups. Oct4-GFP mice were assigned to experiment in Figure 1f according to genotype but randomly allocated into experimental groups. For experiments using wildtype cultured spermatogonia, cells were maintained under the same conditions and randomly assigned to treatment groups.
Blinding	No blinding methods were used as specific procedures and treatments were performed and experimenters needed to be aware of group assignment.

Reporting for specific materials, systems and methods

We require information from authors about some types of materials, experimental systems and methods used in many studies. Here, indicate whether each material, system or method listed is relevant to your study. If you are not sure if a list item applies to your research, read the appropriate section before selecting a response.

Materials & experimental systems

n/a	Involved in the study	
☐	☒	Antibodies
☐	☒	Eukaryotic cell lines
☒	☐	Palaeontology and archaeology
☐	☒	Animals and other organisms
☒	☐	Human research participants
☒	☐	Clinical data
☒	☐	Dual use research of concern

Methods

n/a	Involved in the study	
☒	☐	ChIP-seq
☐	☒	Flow cytometry
☒	☐	MRI-based neuroimaging

Antibodies

Antibodies used	Antibodies used in Immunofluoresence:<br>Goat polyclonal anti-E-Cadherin 1:250 R&D AF748<br>Goat polyclonal anti-GFRa1 1:250 R&D AF560

Goat polyclonal anti-PLZF 1:500 R&D AF2944
Goat polyclonal anti-uPAR 1:100 R&D AF534
Rabbit monoclonal anti-EOMES clone 1219A 1:1000 R&D MAB8889
Antibodies used in Western Blot
Rabbit monoclonal anti-phospho-RPS6 (Ser235/236) clone D57.2.2E 1:300 Cell Signaling Technology #4548
Rabbit monoclonal anti-RARγ clone D3A4 1:500 Cell Signaling Technology #8965
Rabbit monoclonal anti-DDX4/VASA clone D10C5 1:500 Cell Signaling Technology #8761
Rabbit monoclonal anti-FOXO1 clone C29H4 1:500 Cell Signaling Technology #2880
Rabbit monoclonal anti-Smooth Muscle Actin 1:500 Cell Signaling Technology #19245
Rabbit polyclonal anti-Cyclin B1 1:250 Cell Signaling Technology #4138
Mouse monoclonal anti-Myc-Tag clone 9B11 1:200 Cell Signaling Technology #2276
Mouse monoclonal anti-HA-Tag clone 6E2 1:200 Cell Signaling Technology #2367
Rabbit mononclonal anti-Cleaved Caspase-3 (Asp175) clone D3E9 1:200 Cell Signaling Technology #9579
Rabbit polyclonal anti-Cleaved Caspase-9 (Mouse Specific) 1:200 Cell Signaling Technology #9509
Rabbit polyclonal anti-FOXM1 1:500 Proteintech 13147-1-AP
Rabbit monoclonal anti-Cyclin D1 clone SP4 1:250 Abcam ab16663
Rabbit polyclonal anti-SALL4 1:2000 Abcam ab29112
Rat monoclonal anti-KI67 clone SolA15, eBioscienceTM 1:250 Invitrogen 14-5698-82
Rat monoclonal anti-GILZ clone CFMKG15, eBioscienceTM 1:1000 Invitrogen 14-4033-82
Rabbit polyclonal anti-Sox9 1:1000 MERCK AB5535
Secondary antibodies
Alexa Fluor® 647 AffiniPure Donkey Anti-Rat IgG (H+L) 1:500 Jackson Immuno Research 712-605-153
Alexa Fluor® 594 AffiniPure Donkey Anti-Mouse IgG (H+L) 1:500 Jackson Immuno Research 715-585-150
Alexa Fluor® 488 AffiniPure Donkey Anti-Mouse IgG (H+L) 1:500 Jackson Immuno Research 715-545-150
Donkey anti-Rabbit IgG (H+L) Highly Cross-Adsorbed Secondary Antibody, Alexa Fluor 555 1:500 Invitrogen A-31572
Donkey anti-Goat IgG (H+L) Highly Cross-Adsorbed Secondary Antibody, Alexa Fluor Plus 488 1:500 Invitrogen A32814
Antibodies used in Western blot:
Rabbit monoclonal anti-phospho-RPS6 (Ser235/236) clone D57.2.2E 1:2000 Cell Signaling Technology #4858
Rabbit monoclonal anti-RPS6 clone 5G10 1:1000 Cell Signaling Technology #2217
Rabbit monoclonal anti-phospho-ERK1/2 (Thr202/Tyr204) clone D13.14.4E 1:2000 Cell Signaling Technology #8544
Rabbit monoclonal anti-ERK1/2 clone 137F5 1:1000 Cell Signaling Technology #4695
Rabbit monoclonal anti-phospho-AKT (Ser473) clone D9E 1:2000 Cell Signaling Technology #4060
Rabbit monoclonal anti-AKT clone C67E7 1:1000 Cell Signaling Technology #4691
Rabbit monoclonal anti-Survivin clone 71G4B7 1:1000 Cell Signaling Technology #2808
Mouse monoclonal anti-Cyclin B1 clone V152 1:1000 Cell Signaling Technology #4135
Mouse monoclonal anti-b-Actin clone AC-15 1:5000 Sigma A5441
Rabbit polyclonal anti-FOXM1 1:1000 Proteintech 13147-1-AP
Rabbit monoclonal anti-Cyclin D1 clone SP4 1:250 Novus Biologicals NB600-584
Hamster monoclonal anti-PLZF clone 9E12 1:5000 Ref. 23 (PMID: 20691905) Memorial Sloan-Kettering Cancer Center
Mouse monoclonal anti-Myc-Tag clone 9B11 1:1000 Cell Signaling Technology #2276
Mouse monoclonal anti-HA-Tag clone 6E2 1:1000 Cell Signaling Technology #2367
Peroxidase AffiniPure Goat Anti-Rabbit IgG (H+L) 1:1000 Jackson Immuno Research 111-035-144
Peroxidase AffiniPure Goat Anti-Mouse IgG (H+L) 1:1000 Jackson Immuno Research 115-035-003
Peroxidase AffiniPure Goat Anti-Armenian Hamster IgG (H+L) 1:1000 Jackson Immuno Research 127-035-099
Antibodies used in Flow Cytometry:
APC-conjugated anti-E-Cadherin clone DECMA-1 1:250 Biolegend 147311
PE-conjugated anti-E-Cadherin clone DECMA-1 1:250 Biolegend 147303
APC-conjugated anti-c-KIT clone 2B8 1:500 Biolegend 105812
FITC-conjugated anti-integrin a6 clone GoH3 1:250 Biolegend 313605
PE-Cy7-conjugated anti-integrin a6 clone GoH3 1:1000 Invitrogen 25-0495-82
APC-conjugated anti-EpCAM clone G8.8 1:500 Biolegend 118213
PE-Cy7-conjugated anti-EpCAM clone G8.8 1:1000 Biolegend 118216
PE-Cy7-conjugated anti-MCAM clone ME-9F1 1:4000 Biolegend 134713
Goat polyclonal anti-uPAR 1:100 R&D AF534
Alexa Fluor® 647 conjugated hamster monoclonal anti-PLZF clone 9E12 1:2000 Ref 23 (PMID: 20691905) Memorial Sloan-Kettering Cancer Centre

Validation

Antibodies used in Immunofluorescence
Goat polyclonal anti-E-Cadherin 1:250 R&D AF748, validation https://www.rndsystems.com/products/human-mouse-e-cadherin-antibody_af748#product-details. Statement: product tested and validated.
Goat polyclonal anti-GFRa1 1:250 R&D AF560, validation https://www.rndsystems.com/products/rat-gfr-alpha-1-gdnf-r-alpha-1-antibody_af560. Statement: product tested and validated.
Goat polyclonal anti-PLZF 1:500 R&D AF2944, validation https://www.rndsystems.com/products/human-plzf-antibody_af2944. Statement: product tested and validated.
Goat polyclonal anti-uPAR 1:100 R&D AF534, validation https://www.rndsystems.com/products/mouse-upar-antibody_af534. Statement: product tested and validated.
Rabbit monoclonal anti-EOMES clone 1219A 1:1000 R&D MAB8889, validation https://www.rndsystems.com/products/mouse-eomes-antibody-1219a_mab8889. Statement: product tested and validated.
Rabbit monoclonal anti-phospho-RPS6 (Ser235/236) clone D57.2.2E 1:300 Cell Signaling Technology #4548, validation https://www.cellsignal.com/products/primary-antibodies/phospho-s6-ribosomal-protein-ser235-236-d57-2-2e-xp-rabbit-mab/4858. Statement: Antibody Guarantee, in-house validation
Rabbit monoclonal anti-RARγ clone D3A4 1:500 Cell Signaling Technology #8965, validation https://www.cellsignal.com/products/primary-antibodies/rarg1-d3a4-xp-rabbit-mab/8965. Statement: Antibody Guarantee, in-house validation
Rabbit monoclonal anti-DDX4/VASA clone D10C5 1:500 Cell Signaling Technology #8761, validation https://www.cellsignal.com/products/primary-antibodies/ddx4-d10c5-rabbit-mab/8761. Statement: Antibody Guarantee, in-house validation

Rabbit monoclonal anti-FOXO1 clone C29H4 1:500 Cell Signaling Technology #2880, validation https://www.cellsignal.com/products/primary-antibodies/foxo1-c29h4-rabbit-mab/2880. Statement: Antibody Guarantee, in-house validation
Rabbit monoclonal anti-Smooth Muscle Actin 1:500 Cell Signaling Technology #19245, validation https://www.cellsignal.com/products/primary-antibodies/a-smooth-muscle-actin-d4k9n-xp-rabbit-mab/19245. Statement: Antibody Guarantee, in-house validation
Rabbit polyclonal anti-Cyclin B1 1:250 Cell Signaling Technology #4138, validation https://www.cellsignal.com/products/primary-antibodies/cyclin-b1-antibody/4138. Statement: Antibody Guarantee, in-house validation
Mouse monoclonal anti-Myc-Tag clone 9B11 1:200 Cell Signaling Technology #2276, validation https://www.cellsignal.com/products/primary-antibodies/myc-tag-9b11-mouse-mab/2276. Statement: Antibody Guarantee, in-house validation
Mouse monoclonal anti-HA-Tag clone 6E2 1:200 Cell Signaling Technology #2367, validation https://www.cellsignal.com/products/primary-antibodies/ha-tag-6e2-mouse-mab/2367. Statement: Antibody Guarantee, in-house validation
Rabbit mononclonal anti-Cleaved Caspase-3 (Asp175) clone D3E9 1:200 Cell Signaling Technology #9579, validation https://www.cellsignal.com/products/primary-antibodies/cleaved-caspase-3-asp175-d3e9-rabbit-mab/9579. Statement: Antibody Guarantee, in-house validation
Rabbit polyclonal anti-Cleaved Caspase-9 (Mouse Specific) 1:200 Cell Signaling Technology #9509, validation https://www.cellsignal.com/products/primary-antibodies/cleaved-caspase-9-asp353-antibody-mouse-specific/9509. Statement: Antibody Guarantee, in-house validation
Rabbit polyclonal anti-FOXM1 1:500 Proteintech 13147-1-AP, validation https://www.ptglab.com/products/FOXM1-Antibody-13147-1-AP.htm#tested-applications. Statement: knockdown/knockout validated
Rabbit monoclonal anti-Cyclin D1 clone SP4 1:250 Abcam ab16663, validation https://www.abcam.com/cyclin-d1-antibody-sp4-ab16663.html. Statement: knockout validated.
Rabbit polyclonal anti-SALL4 1:2000 Abcam ab29112, validation https://www.abcam.com/sall4-antibody-ab29112.html?productWallTab=ShowAll. Knockout validated in PMID 28867346
Rat monoclonal anti-KI67 clone SolA15, eBioscienceTM 1:250 Invitrogen 14-5698-82, validation https://www.thermofisher.com/antibody/product/Ki-67-Antibody-clone-SolA15-Monoclonal/14-5698-82. Statement: advanced verification
Rat monoclonal anti-GILZ clone CFMKG15, eBioscienceTM 1:1000 Invitrogen 14-4033-82, validation https://www.thermofisher.com/antibody/product/GILZ-Antibody-clone-CFMKG15-Monoclonal/14-4033-82. Knockout validated in PMID: 30126904.
Rabbit polyclonal anti-Sox9 1:1000 MERCK AB5535, validation https://www.sigmaaldrich.com/AU/en/product/mm/ab5535?gclid=Cj0KCQiAjc2QBhDgARIsAMc3SqQCyQew-1dHE8iwyVV8y7N0RJH1ZSg2VnAUa5QtdI8d2ALc8uYtfKAaAjfkEALw_wcB. Statement: This highly published antibody has been validated in IHC & WB.
Antibodies used in Western Blot:
Rabbit monoclonal anti-phospho-RPS6 (Ser235/236) clone D57.2.2E 1:2000 Cell Signaling Technology #4858, validation https://www.cellsignal.com/products/primary-antibodies/phospho-s6-ribosomal-protein-ser235-236-d57-2-2e-xp-rabbit-mab/4858. Statement: Antibody Guarantee, in-house validation
Rabbit monoclonal anti-RPS6 clone 5G10 1:1000 Cell Signaling Technology #2217, validation https://www.cellsignal.com/products/primary-antibodies/s6-ribosomal-protein-5g10-rabbit-mab/2217. Statement: Antibody Guarantee, in-house validation
Rabbit monoclonal anti-phospho-ERK1/2 (Thr202/Tyr204) clone D13.14.4E 1:2000 Cell Signaling Technology #8544, validation https://www.cellsignal.com/products/antibody-conjugates/phospho-p44-42-mapk-erk1-2-thr202-tyr204-d13-14-4e-xp-rabbit-mab-hrp-conjugate/8544?_=1645512145692&Ntt=8544&tahead=true. Statement: Antibody Guarantee, in-house validation
Rabbit monoclonal anti-ERK1/2 clone 137F5 1:1000 Cell Signaling Technology #4695, validation https://www.cellsignal.com/products/primary-antibodies/p44-42-mapk-erk1-2-137f5-rabbit-mab/4695. Statement: Antibody Guarantee, in-house validation
Rabbit monoclonal anti-phospho-AKT (Ser473) clone D9E 1:2000 Cell Signaling Technology #4060, validation https://www.cellsignal.com/products/primary-antibodies/phospho-akt-ser473-d9e-xp-rabbit-mab/4060. Statement: Antibody Guarantee, in-house validation
Rabbit monoclonal anti-AKT clone C67E7 1:1000 Cell Signaling Technology #4691, validation https://www.cellsignal.com/products/primary-antibodies/akt-pan-c67e7-rabbit-mab/4691. Statement: Antibody Guarantee, in-house validation
Rabbit monoclonal anti-Survivin clone 71G4B7 1:1000 Cell Signaling Technology #2808, validation https://www.cellsignal.com/products/primary-antibodies/survivin-71g4b7-rabbit-mab/2808. Statement: Antibody Guarantee, in-house validation
Mouse monoclonal anti-Cyclin B1 clone V152 1:1000 Cell Signaling Technology #4135, validation https://www.cellsignal.com/products/primary-antibodies/cyclin-b1-v152-mouse-mab/4135?_=1645512296772&Ntt=4135&tahead=true. Statement: Antibody Guarantee, in-house validation
Mouse monoclonal anti-b-Actin clone AC-15 1:5000 Sigma A5441, validation in PMID 28867346
Rabbit polyclonal anti-FOXM1 1:1000 Proteintech 13147-1-AP, validation https://www.ptglab.com/products/FOXM1-Antibody-13147-1-AP.htm#tested-applications. Statement: knockdown/knockout validated
Rabbit monoclonal anti-Cyclin D1 clone SP4 1:250 Novus Biologicals NB600-584, validation https://www.novusbio.com/products/cyclin-d1-antibody-sp4_nb600-584. Statement: product tested and validated
Hamster monoclonal anti-PLZF clone 9E12 1:5000 Memorial Sloan-Kettering Cancer Center, validation in PMID: 20691905
Mouse monoclonal anti-Myc-Tag clone 9B11 1:1000 Cell Signaling Technology #2276, validation https://www.cellsignal.com/products/primary-antibodies/myc-tag-9b11-mouse-mab/2276. Statement: Antibody Guarantee, in-house validation
Mouse monoclonal anti-HA-Tag clone 6E2 1:200 Cell Signaling Technology #2367, validation https://www.cellsignal.com/products/primary-antibodies/ha-tag-6e2-mouse-mab/2367. Statement: Antibody Guarantee, in-house validation
Antibodies for flow cytometry:
APC-conjugated anti-E-Cadherin clone DECMA-1 1:250 Biolegend 147311, validation https://www.biolegend.com/en-us/products/apc-anti-mouse-human-cd324-e-cadherin-antibody-16412. Statement: FC-Quality tested
PE-conjugated anti-E-Cadherin clone DECMA-1 1:250 Biolegend 147303, validation https://www.biolegend.com/en-us/products/pe-anti-mouse-human-cd324-e-cadherin-antibody-9276. Statement: FC-Quality tested
APC-conjugated anti-c-KIT clone 2B8 1:500 Biolegend 105812, validation https://www.biolegend.com/en-us/products/apc-anti-mouse-cd117-c-kit-antibody-72. Statement: FC-Quality tested
FITC-conjugated anti-integrin a6 clone GoH3 1:250 Biolegend 313605, validation https://www.biolegend.com/en-us/products/fitc-anti-human-mouse-cd49f-antibody-2606. Statement: FC-Quality tested
PE-Cy7-conjugated anti-integrin a6 clone GoH3 1:1000 Invitrogen 25-0495-82, validation https://www.thermofisher.com/antibody/product/CD49f-Integrin-alpha-6-Antibody-clone-eBioGoH3-GoH3-Monoclonal/25-0495-82. Statement: flow cytometry tested
APC-conjugated anti-EpCAM clone G8.8 1:500 Biolegend 118213, validation https://www.biolegend.com/en-us/products/apc-anti-mouse-cd326-ep-cam-antibody-4974. Statement: FC-Quality tested
PE-Cy7-conjugated anti-EpCAM clone G8.8 1:1000 Biolegend 118216, validation https://www.biolegend.com/en-us/products/pe-cyanine7-anti-mouse-cd326-ep-cam-antibody-5303. Statement: FC-Quality tested

PE-Cy7-conjugated anti-MCAM clone ME-9F1 1:4000 Biolegend 134713, validation https://www.biolegend.com/en-us/products/pe-cyanine7-anti-mouse-cd146-antibody-9322. Statement: FC-Quality tested
Goat polyclonal anti-uPAR 1:100 R&D AF534, validation https://www.rndsystems.com/products/mouse-upar-antibody_af534/. Statement: product tested and validated.
Alexa 647 Hamster monoclonal anti-PLZF clone 9E12 1:2000 Memorial Sloan-Kettering Cancer Center, validation in PMID: 20691905

Eukaryotic cell lines

Policy information about cell lines

Cell line source(s)	293HEK cells (originally from ATCC) were used for production of lentivirus.
Authentication	No additional authentication of 293HEK line was performed.
Mycoplasma contamination	No additional testing for mycoplasma was performed.
Commonly misidentified lines (See ICLAC register)	None.

Animals and other organisms

Policy information about studies involving animals; ARRIVE guidelines recommended for reporting animal research

Laboratory animals	Adult wildtype male mice were of C57BL6 background and 6-8 weeks old. Adult Oct4-GFP transgenic male mice (6-8 weeks old) were of C57BL6/ CBA/129T2svJ mixed background and previously described (PMID: 30026551). Mice were housed at 18-24 degrees Celsius with 40-70% humidity and a 12-hour day-night cycle.
Wild animals	This study did not use wild animals.
Field-collected samples	This study did not use samples collected from the field.
Ethics oversight	Animal studies were performed in accordance with the Australian Code of Practice for the Care and Use of Animals for Scientific Purposes. Experiments were subject to approval by the Monash University and Medical Centre Animal Ethics Committees (Projects MARP-2015-025 and MMCB-2020-15).

Note that full information on the approval of the study protocol must also be provided in the manuscript.

Flow Cytometry

Plots

Confirm that:

☒ The axis labels state the marker and fluorochrome used (e.g. CD4-FITC).

☒ The axis scales are clearly visible. Include numbers along axes only for bottom left plot of group (a 'group' is an analysis of identical markers).

☒ All plots are contour plots with outliers or pseudocolor plots.

☒ A numerical value for number of cells or percentage (with statistics) is provided.

Methodology

Sample preparation	For analysis and sorting of live testis cells (PMID: 30126904): Decapsulated and minced adult testes were washed in phosphate-buffered saline (PBS) to remove spermatozoa and debris. A single cell suspension was then generated by digestion of tubules with 1mg/ml type II collagenase (Sigma) in un-supplemented DMEM (Thermo) with 40 U/ml DNase I at 37 degrees Celsius for 10 minutes with occasional agitation. Cells were dissociated in PBS with 2% fetal bovine serum (FBS), put through a 70 micron cell strainer and washed in PBS prior to use. Harvested cells were stained for 25 minutes on ice with antibodies in PBS with 2% FBS.<br>For analysis of fixed and permeabilised testis cells (PMID: 28867346): Single cell suspensions were generated from washed testis tubules by sequential digest first in 1mg/ml type IV collagenase (Sigma) in un-supplemented DMEM (Thermo) with 40 U/ml DNase I at 37 degrees Celsius for 10 minutes with occasional agitation then in 0.25% Trypsin (Thermo) in PBS with 40 U/ml DNase I at 37 degrees Celsius for 5 minutes . Cells were dissociated in PBS with 10% fetal bovine serum (FBS), put through a 70 micron cell strainer and washed prior to use. Harvested cells were stained for 30 minutes at room temperature with antibodies in PBS with 2% FBS.
Instrument	Cells were sorted with an Influx Cell Sorter (BD Biosciences) and analyzed using an LSR Fortessa X-20 (BD Biosciences).
Software	Data processed with FlowJo software v8.7
Cell population abundance	For FACS sorting experiments, purity of isolated spermatogonia was approx. 95% as determined by single cell RNA-Seq analysis (Supplementary Table 7).
Gating strategy	For analysis and sorting, a FSC/SSC gate was used to exclude cell debris and clumps (approx. 70% cells retained). Doublets

Gating strategy

were excluded based on FSC-H vs. FSC-A. For live cells, a viability gate (DAPI) was used to exclude dead cells. This population was then gated as indicated in individual figures. Positive and negative gates were set according to relevant isotype or secondary antibody-stained control samples.

☒ Tick this box to confirm that a figure exemplifying the gating strategy is provided in the Supplementary Information.
